# *Omphalotus yunnanensis*: A New Poisonous Mushroom Discovered from China Based on Morphological, Molecular and Toxin-Detection Evidence

**DOI:** 10.3390/toxins18010040

**Published:** 2026-01-12

**Authors:** Zhong-Feng Li, Jing Zhang, Xiang-Dong Min, Hong-Shun Zhang, Li Chen, Dai-Neng Li, Yi-Zhe Zhang, Ming-Xuan Yuan, Zhi-Yuan Liu, Jia-Ju Zhong, Meng-Huan Ruan, Hai-Jiao Li

**Affiliations:** 1 State Key Laboratory of Trauma and Chemical Poisoning, National Institute of Occupational Health and Poison Control, Chinese Centre for Disease Control and Prevention, No. 29 Nanwei Road, Xicheng District, Beijing 100050, China; i1175cayy@163.com (Z.-F.L.); zhanghs@niohp.chinacdc.cn (H.-S.Z.); zyz97@263.net (Y.-Z.Z.); yuanmingxuan1120@163.com (M.-X.Y.); liuzhiyuan000529@163.com (Z.-Y.L.); zhongjiaju1990@126.com (J.-J.Z.); ruanmenghuan0205@163.com (M.-H.R.); 2 Beijing Municipal Center for Disease Control and Prevention, No. 16 Hepingli Middle Street, Dongcheng District, Beijing 100013, China; brightjing@163.com; 3 Yunnan Center for Disease Control and Prevention (Yunnan Academy of Preventive Medicine), No. 1177 Xianghe Street, Chenggong District, Kunming 650022, China; mingxiangdong@126.com; 4 Honghe Hani and Yi Autonomous Prefecture Center for Disease Control and Prevention, Guanlan Road, Mengzi City, Honghe 661199, China; hhgjchenli@163.com; 5 Jinping Miao-Yao-Dai Autonomous County Center for Disease Control and Prevention, No. 249 Hedongnan Road, Jinhe Town, Jinping County, Honghe 661100, China; jpwsjyzk@163.com

**Keywords:** new taxon, mushroom poisoning, mycotoxins, phylogeny, UPLC-MS/MS

## Abstract

In the past few years, several mushroom poisoning incidents caused by *Omphalotus* species have occurred in China. In addition to *O. guepiniformis* and *O. olearius*, a new white *Omphalotus* species, *O. yunnanensis*, was discovered in Southwestern and Southern China based on morphological, molecular and toxin-detection evidence. *Omphalotus yunnanensis* is characterized by its small, cream to white basidiomata with a hygrophanous pileal surface, non-bioluminescent lamellae, broadly ellipsoid to subglobose basidiospores (8–12.5 × 7–10 μm), fusoid to ventricose cheilocystidia with occasional apical outgrowths, cream to white pileipellis composed of thick-walled, subsoil to solid hyphae, clavate, and fusoid to ventricose caulocystidia with occasional apical outgrowths. The species has been discovered in tropical to subtropical areas in Southwestern and Southern China. Phylogenetic analyses based on ITS and nrLSU showed that the new species clustered with the Australasian species *O. nidiformis*, but can be easily distinguished by its smaller, white to cream pileus, non-bioluminescent lamellae, larger basidiospores and growing on Fagaceae species. Illudin S was detected in this new species using UPLC-MS/MS, at 6.98 to 86.1 mg/kg of the content (dry weight), while no illudin M was detected.

## 1. Introduction

The genus *Omphalotus* Fayod (Omphalotaceae, Agaricales) comprises saprotrophic or weakly parasitic wood-decaying fungi, characterized by distinct morphological characters: funnel-shaped to flabelliform basidiomata; decurrent, luminescent (or not) lamellae; white to pale-colored; ellipsoid; globose to subglobose; thin- to thick-walled; glabrous to finely rough basidiospores; without pleurocystidia; and with cheilocystidia often bearing apical outgrowths and hyphae with clamp connections [1]. The genus includes nearly ten recognized species, four of which have been recorded in China: *O. flagelliformis* Zhu L. Yang & B. Feng (a new species described in 2013), *O. guepiniformis* (Berk.) Neda (syn. *O. japonicus*), *O. mangensis* (Jian Z. Li & X.W. Hu) Kirchm. & O.K. Mill., and *Lampteromyces luminescens* M. Zang, which is closely related to *O. guepiniformis* and has uncertain generic placement [2]. These species are distributed across tropical to subtropical and temperate regions of China, and are predominantly associated with the decaying wood of Fagaceae and other deciduous trees [3]. The genus *Omphalotus* has a global distribution, with species reported from Europe, North America, Australia, and East Asia [4]. However, tropical and subtropical populations of *Omphalotus* remain understudied, with limited data on their taxonomic status and toxin profiles.

Mushroom poisoning is a global public health concern, with an estimated 10,000–50,000 cases annually worldwide, and misidentification of toxic species as edible ones is a common global risk factor [5]. Thus, the discovery of *O. yunnanensis* and its toxin characteristics provide critical data for global fungal diversity databases, inform international taxonomic revisions of *Omphalotus*, and offer a model for integrating morphology, phylogeny, and toxinology in identifying new toxic fungi—relevant to mycologists, toxicologists, and public health practitioners worldwide [6].

*Omphalotus*-caused mushroom poisoning has become a notable public health problem in China. According to surveillance data from 2021 to 2024, *Omphalotus* species (including *O. guepiniformis*, *O. olearius*, and the new species described below) were responsible for 2–7 poisoning incidents annually, involving 3–44 patients per year [3]. Geographically, these incidents were concentrated in Southwest China (Yunnan, Guizhou, and Sichuan) and Central-South China (Hunan, Guangxi, and Hainan). Clinical manifestations largely involve acute gastroenteritis, with symptoms including nausea, vomiting, abdominal cramps, and diarrhea occurring 1–6 h post-ingestion [3,7]. The primary cause of poisoning is the misidentification of *Omphalotus* species as edible mushrooms, e.g., *Pleurotus* spp. and *Cantharellus* spp., due to superficial morphological similarities, especially in rural areas [7].

The toxins of *Omphalotus* species are illudins, a kind of cytotoxic sesquiterpenoid [8,9,10,11,12]. Two major congeners, illudin S and illudin M, were first isolated from *O. illudens* (Schwein.) Bresinsky et Besl., and later discovered in *O. olearius*, *O. guepiniformis*, and *O. nidiformis* (Berk.) O.K. Mill [9,11,13]. Illudins exert their toxicity by alkylating DNA and inhibiting eukaryotic DNA replication, leading to intestinal epithelial cell damage and subsequent gastroenteritis [10,12]. Analytical studies using liquid chromatography–tandem mass spectrometry (LC-MS/MS) and ultra-performance liquid chromatography–tandem mass spectrometry (UPLC-MS/MS) have confirmed illudin S as the primary toxic component in Asian *Omphalotus* species, while illudin M is either absent or present at undetectable levels in most Chinese isolates [10,11,12]. This toxin profile is consistent across the genus, making illudins a reliable chemotaxonomic marker for *Omphalotus* [2,10].

Recent poisoning surveillance has uncovered a series of unreported outbreaks caused by a morphologically distinct white *Omphalotus* species, which was initially recorded as *Omphalotus* sp. in epidemiological reports [6,7]. According to incident records from 2022 to 2025, this species has caused four confirmed poisoning events in China: (1) Jinping County, Yunnan (September 2022, 1 patient): nausea, violent vomiting, and abdominal cramps 2 h post-ingestion, symptom resolution within 48 h; (2) Hekou County, Yunnan (July 2023, 2 patients): watery diarrhea (6–8 episodes/day), abdominal colic, and mild dehydration 3 h post-ingestion, treated with rehydration therapy and recovered in 72 h; (3) Honghe County, Yunnan (October 2023, 6 patients): collective poisoning after consuming a white *Omphalotus* species misidentified as *Pleurotus* spp., presenting with nausea, vomiting, and crampy abdominal pain (latency 1.5–4 h), no hospitalizations required; (4) Wuzhishan City, Hainan (January 2025, 5 patients): severe abdominal spasms, vomiting, and diarrhea lasting 36 h. Illudin S alkylates DNA at N3 of adenine and N7 of guanine, inhibiting eukaryotic DNA replication and inducing apoptosis in intestinal epithelial cells. A multicenter study of *Omphalotus* poisoning in China reported that an illudin S intake of ≥0.05 mg/kg body weight (corresponding to 3 mg for a 60 kg adult) induces acute gastroenteritis. For the new species, ingestion of 10 g dried mushroom (a typical serving) results in an illudin S intake of 0.0698–0.861 mg, which exceeds the toxicity threshold—consistent with the clinical symptoms observed in the four poisoning incidents. No illudin M was detected, aligning with the toxin profile of Asian *Omphalotus* species [11,12].

The aims of this study were therefore (1) to determine the taxonomic status of these unique mushrooms through a critical examination of their morphological characteristics, along with a phylogenetic analysis based on internal transcribed spacer (ITS) sequences and the nuclear large subunit (nrLSU) of ribosomal DNA, and (2) to conduct a comprehensive toxin profiling using ultra-performance liquid chromatography–tandem mass spectrometry (UPLC-MS/MS) to screen for a wide array of mushroom toxins, with a particular focus on illudins S and M. By integrating morphological, phylogenetic, and chemotaxonomic evidence, we describe a new species, *Omphalotus yunnanensis*, in detail.

## 2. Results

### 2.1. Molecular Phylogeny

**Figure 1:**
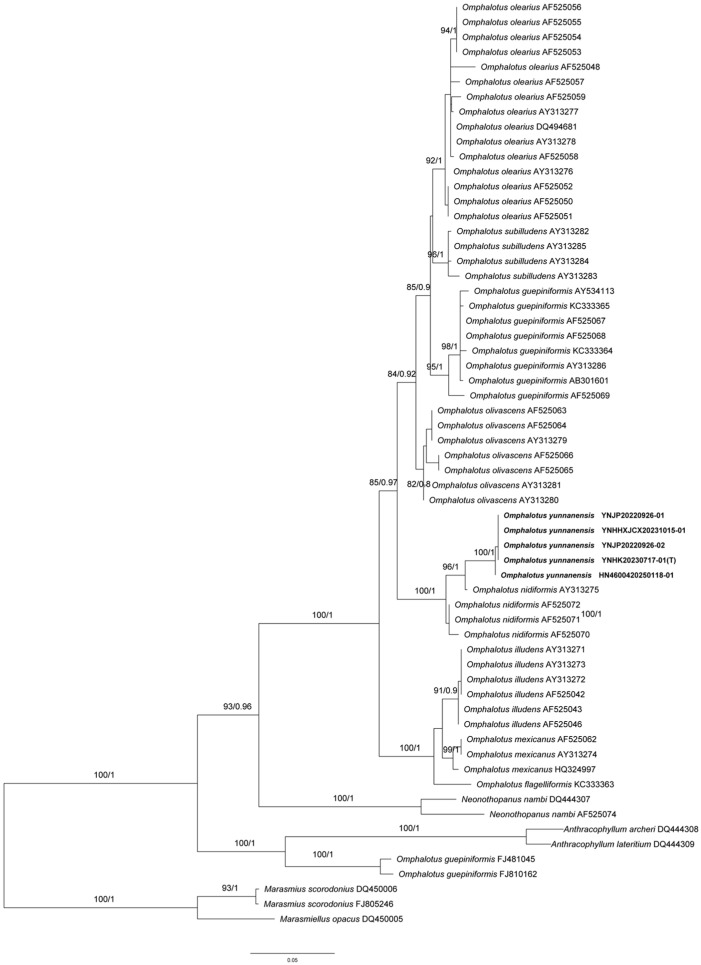
Phylogenetic tree of *Omphalotus* and related genera based on combined ITS sequence data. The tree was constructed using Maximum Likelihood analysis. Bootstrap values (ML, ≥80%) and Bayesian posterior probabilities (≥0.8) are shown at the nodes. Note: T means holotype.

jModelTest (ModelTest-NG v0.1.7) suggested HKY + I + G as the best-fit nucleotide evolution models for the dataset. The average standard deviation of BI split frequencies was 0.002327 at the end of the run. BI analyses resulted in almost identical tree topologies to the ML analysis. The ML tree is provided in Figure 1, with the likelihood bootstrap values (≥50%, first) and Bayesian posterior probabilities (≥0.95, second) labeled along the branches. We conducted phylogenetic analyses of the ITS dataset using Maximum Likelihood (ML) and Bayesian Inference (BI) methods to establish the taxonomic position of the newly collected *Omphalotus* specimens. Consistent with the findings of Yang & Feng [2] and Kirchmair et al. [13], the genus *Omphalotus* formed a well-supported monophyletic clade (ML = 100%, BI = 1.00) in both phylogenetic trees [14].

Five specimens collected from Yunnan and Hainan Province formed a distinct monophyletic clade within *Omphalotus*, with maximum statistical support (ML = 100%, BI = 1.00, Figure 1). This clade was clearly separated from other recognized *Omphalotus* species.

The new species clustered with one sequence (AY313275) of *Omphalotus nidiformis* (ML = 96%, BI = 1.00, Figure 1), before grouping with another three *O. nidiformis* sequences (AF525070–AF525072, ML = 100%, BI = 1.00, Figure 1).

### 2.2. Taxonomy and Morphology

#### *Omphalotus yunnanensis* Hai J. Li & Zhong-Feng Li, sp. nov. Figure 2 and Figure 3

**MycoBank**: 861469

**Diagnosis**: *Omphalotus yunnanensis* is characterized by its semicircular, flabelliform to infundibuliformis, cream to white basidiomata with a hygrophanous pileal surface, non-bioluminescent lamellae, large, broadly ellipsoid to subglobose basidiospores (8–12.5 × 7–10 μm), fusoid to ventricose cheilocystidia which sometimes with apical outgrowths, cream to white pileipellis composed with thick-walled, subsoil to solid hyphae, clavate, fusoid to ventricose caulocystidia which sometimes with apical outgrowths, and discovered from tropical to subtropical Southwestern and Southern areas of China.

**Holotypus**: CHINA, Yunnan Province, Honghe Hani and Yi Autonomous Prefecture, Hekou Yao Autonomous County, Lianhuatan Town, Zhonglinggang Village, Yaomaji Group 1, on fallen angiosperm trunk, 17 July 2023, *YNHK20230717-01* (*BJFC054640*, ITS sequence accession number: PX596399, LSU sequence accession number: PX588451).

**Etymology**: The specific epithet “yunnanensis” refers to Yunnan Province, China, the type locality of the new species.

**Macrostructures**: Pileus 30–80 mm latus, semicircular, flabelliform to infundibuliformis, convex when young, becoming applanate with a depressed center at maturity; cream to white, hygrophanous when fresh, glabrous; margin entire, slightly incurved when fresh. Lamellae decurrent, crowded, cream to white, not bioluminescent. Stipe 30–70 × 5–15 mm, lateral, eccentric to central, subcylindric, slightly attenuate ·towards the base; surface concolorous with the pileus or paler, upper part glabrous and velutinate near the base. Context solid, cream to white, tough. Odor indistinct; taste not tested.

**Figure 2:**
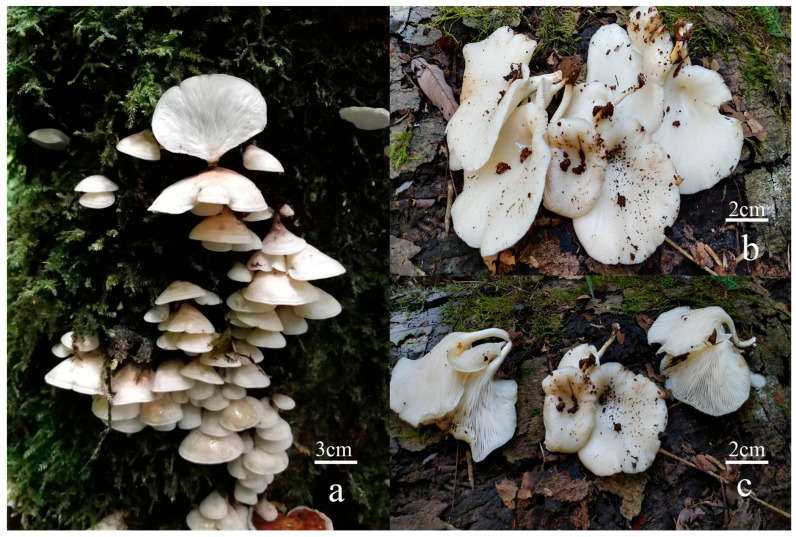
Basidiomata of *Omphalotus yunnanensis*. (**a**): YNHK20230717-01 (holotype). (**b**,**c**): YNJP20220926-01.

**Figure 3:**
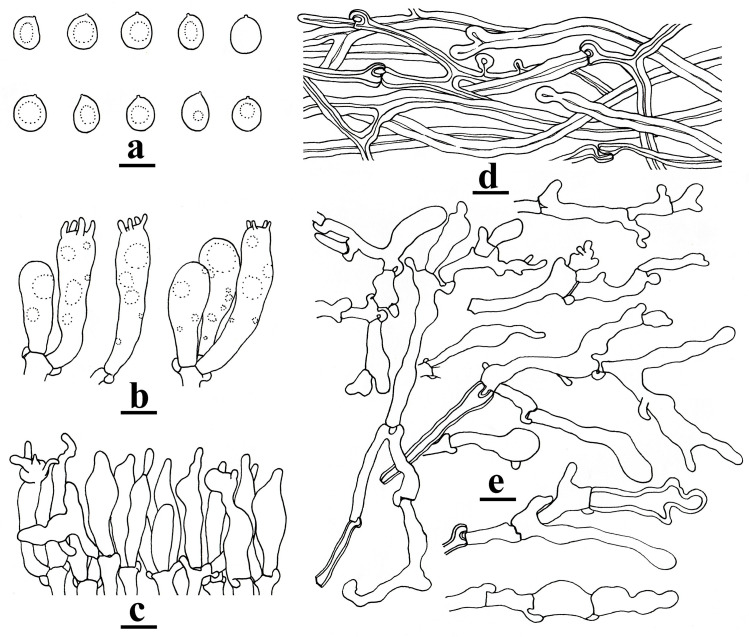
Microstructures of *Omphalotus yunnanensis* (YNHK20230717-01, holotype). (**a**). Basidiospores; (**b**). basidia; (**c**). cheilocystidia; (**d**). pileipellis hyphae; (**e**). caulocystidia. Bars = 10 μm. Figure drawn by H.-J. Li.

**Microstructure**: Basidiospores [60/5/3] (7.2–)8–12.5(–13) × (6.7–)7–10(–11.6) μm, averaging 10.37 × 8.65 μm, Q = (1.05–)1.07–1.36(–1.44), Q_m_ = 1.20 ± 0.10, mostly broadly ellipsoid to subglobose, rarely ellipsoid, nearly colorless to hyaline, mostly guttulate, smooth, thin-walled to slightly thick-walled (≤0.5 μm thick), non-amyloid, non-dextrinoid (Figure 3a). Basidia 25–35 × 7–9 μm, clavate, hyaline, 4-spored, with clamp connections at the base (Figure 3b). Cheilocystidia abundant, 20–35 × 6–8 μm, flexuous, mostly fusoid to ventricose, sometimes with rostrate to mucronate apical outgrowths, rarely with secondary septum, colorless and hyaline, thin-walled (Figure 3c). Pleurocystidia absent. Lamellar trama composed of ± regularly arranged thin- to slightly thick-walled (≤0.5 μm thick) filamentous hyphae, 4–10 μm wide. Pileipellis a cutis composed of repent hyphae, 3–8 μm wide, cream to white, thick-walled, subsoil to solid (Figure 3d). Stipitipellis consisting of vertically arranged, branching and sometimes anastomosing hyphae, yellowish to yellowish, thick-walled, 5–9 μm wide; Caulocystidia clavate, fusoid to ventricose, sometimes with rostrate to mucronate apical outgrowths, occasionally with secondary septum, nearly colorless and hyaline, mostly thin-walled, rarely thick-walled, 25–40 × 5–7 μm (Figure 3e).

**Additional specimen examined**: CHINA, Yunnan Province, Honghe Prefecture, Jinping County, Jinshuihe Township, Pujiao Village, 24 September 2022, *YNJP20220926-01* (ITS sequence accession number: PX596400) and *YNJP20220926-02* (ITS sequence accession number: PX596398); Jiache Town, Hemo Village, 14 October 2023, *YNHHXJCX20231015-01* (ITS sequence accession number: PX588448, LSU sequence accession number: PX588454); Hekou County, Lianhuatan Township, Zhonglinggang Village, Yaomaji Group 1, 17 July 2023, *YNHK20230717-01* (ITS sequence accession number: PX596399, LSU sequence accession number: PX588451); Hainan Province, Wuzhishan, Shuiman Township, Xincun Group 1, 18 January 2025, *HN4600420250118-01* (ITS sequence accession number: PX588449, LSU sequence accession number: PX588453).

### 2.3. Toxin Detection Results

Linearity: All target toxins showed good linearity in the range of 0.5–200 μg/L, with correlation coefficients (R^2^) > 0.995 (e.g., R^2^ = 0.998 for illudin S) [10,15]. Limits of Detection (LODs) and Quantification (LOQs): LODs ranged from 2.0 to 500.0 mg/kg, and LOQs ranged from 5.0 to 500 mg/kg (LOD = 3.0 mg/kg, LOQ = 5 mg/kg for illudin S). Recovery and Precision: The mean recoveries at three spiking levels (10, 20, 100 μg/kg) were 78.6–109.7%, with relative standard deviations (RSDs) ≤ 9.0% (*n* = 6), thus meeting the mycotoxin detection requirements [16,17]. Only illudin S was detected in all three *O. yunnanensis* specimens, with its content ranging from 6.98 to 86.1 mg/kg (dry weight, Table 1); no other target toxins were found.

## 3. Discussion

This study integrates morphological observation, phylogenetic analysis, and toxin detection to address two core objectives: delimiting the taxonomic status of a novel *Omphalotus* species from China and characterizing its toxin profile. The findings enrich our understanding of the fungal diversity of *Omphalotus*, providing critical data for assessing the public health risks posed by poisonous mushrooms.

ITS sequence-based phylogenetic reconstruction confirmed that the five specimens from Yunnan and Hainan formed a strongly supported monophyletic clade (ML = 100%, BI = 1.00), and one that is clearly distinct from all known *Omphalotus* species. The new species grouped with the Australasian species *O. nidiformis*, but can be easily distinguished by its smaller (30–80 mm vs. 50–250 mm in diameter), white to cream pileus, non-bioluminescent lamellae, larger basidiospores (8–12.5 × 7–10 μm vs. 6–8 × 4–5 μm) and its growing on Fagaceae species vs. on *Eucalyptus* spp. and *Acacia* spp., as is seen for *O. nidiformis* [16,17,18].

Morphologically speaking, *Omphalotus guepiniformis* can be distinguished from the new species by its larger, pleurotoid to fan-shaped basidiomata, red-brown, blue to violet pileus surface, bioluminescent lamellae, larger, nearly colorless and hyaline or yellowish to brownish, thin- to thick-walled basidiospores (10–15.5 × 9.5–14.5 μm), with a smooth surface when thin-walled and finely rough to verrucous when thick-walled [2,11,19].

*Omphalotus mangensis*, originally described in Hunan, China, also produces white basidiomata, which can be easily differentiated from *O. yunnanensis* by its pleurotoid to fan-shaped basidiomata, bioluminescent lamellae, larger, globose to subglobose, thin- to thick-walled basidiospores (11–16 × 10–15 μm) [2].

This new species can be distinguished from the widespread *O. olearius* (distributed in Europe and introduced into East Asia [5]) by its white to cream pileus surface, non-bioluminescent lamellae and larger basidiospores (8–12.5 × 7–10 μm vs. 7–10 × 5–7 μm) [9,11,20].

UPLC-MS/MS detection showed that *O. yunnanensis* produces illudin S, without illudin M, alongside the well-documented trait that “illudins are characteristic sesquiterpenoid toxins of the *Omphalotus* genus” [9,10]. This finding reinforces chemotaxonomic consistency within *Omphalotus*: previous studies have confirmed illudins in *O. olearius* (Europe), *O. guepiniformis* (East Asia), and *O. nidiformis* (Australia) [8,10,11], and the presence of these toxins in *O. yunnanensis* further validates these compounds as a synapomorphy (shared derived trait) of the genus [21].

Interspecific comparison of illudin S concentrations reveals that *O. yunnanensis* (6.98–86.1 mg/kg dry weight) falls within the toxic range of other *Omphalotus* species. For *O. guepiniformis*, illudin S concentrations were 12.3–78.5 mg/kg dry weight [22]; for *O. olearius*, concentrations ranged from 8.7–92.4 mg/kg dry weight [23]; and for *O. nidiformis*, illudin S was 5.6–68.9 mg/kg dry weight [10]. The overlapping concentration ranges explain the consistent acute gastroenteritis symptoms induced by these species, confirming that *O. yunnanensis* shares the same toxicological profile as its congeners [24].

## 4. Conclusions

Based on systematic morphological, phylogenetic, and chemotaxonomic investigations, we described a new poisonous mushroom species, *Omphalotus yunnanensis*.

## 5. Materials and Methods

### 5.1. Sample Collection and Morphological Observation

All specimens in this study were obtained from epidemiological surveys of mushroom poisoning incidents involving fungi collected from tropical to subtropical evergreen broad-leaved forests. Specimen collection and identification followed the standard methods for taxonomic research [17,25,26,27].

In situ field photographs of the fruiting bodies were taken to record their macroscopic morphological characteristics, including pileus color, lamella arrangement, stipe features, and bioluminescence. The fresh specimens were dried in a 45 °C blast drying oven and then preserved in the Herbarium of the National Institute of Occupational Health and Poison Control, Chinese Center for Disease Control and Prevention.The holotype was also preserved in the herbarium of Beijing Forestry University (BJFC).

Microscopic structural observation followed the established technical protocols [2,26]. After rehydrating the dried specimens, free-hand sections were prepared. Tissues from the pileipellis, lamellae, and stipe context were mounted in 5% potassium hydroxide (KOH) solution, and all microscopic characteristics were observed and measured using a Nikon E 80I microscope (Nikon Corporation, Tokyo, Japan). In the basidiospore descriptions, the abbreviation [n/m/p] means n basidiospores measured from m basidiomata of p collections; Q is used to mean “length/width ratio” of a spore in side view, while Q_m_ means average Q of all basidiospores ± sample standard deviation.

### 5.2. DNA Extraction, PCR Amplification and Phylogenetic Analysis

The Phire^®^ Plant Direct PCR Kit (Finnzymes Oy, Espoo, Finland) was used to obtain PCR products from dried specimens, according to the manufacturer’s instructions with some modifications [28]. ITS5/ITS4 was used to amplify the internal transcribed spacer (ITS) regions [29], while LR0R/LR5 was used to amplify the nuclear large subunit rDNA (LSU) regions [30]. For the PCR procedures, we followed the method presented by Tang et al. [28]. All newly generated sequences in this study were deposited in GenBank (PX588448-PX588449, PX596398-PX596400, PX588451, PX588453-PX588454).

Reference sequences of Omphalotaceae and related taxa were downloaded from GenBank. Maximum likelihood (ML) analyses and Bayesian inference (BI) were carried out using RAxML v.8.2.10 [31] and MrBayes 3.2.6 [15]. In ML analysis, statistical support values were obtained using rapid bootstrapping with 1000 replicates, with default settings used for other parameters. For BI, the best-fit substitution model was estimated with jModeltest v.2.17. Four Markov chains were run 4,000,000 times for the dataset until the split deviation frequency value was lower than 0.01. Trees were sampled every 100th generation. The first quarter of these, representing the analysis burn-in phase, were discarded, and the remaining trees were used to calculate posterior probabilities (BPP) in the majority rule consensus tree [27]. *Marasmius scorodonius* and *Marasmiellus opacus* were selected as outgroups [1].

### 5.3. Toxin Analysis

#### 5.3.1. Sample Preparation

Sample preparation was performed according to established protocols with minor modifications [10,12]. Dried basidiomata were finely homogenized using a laboratory mill. Approximately 10 mg of the homogenized sample was accurately weighed into a 15 mL polypropylene centrifuge tube. Extraction was carried out with 2 mL of methanol–water (70:30, *v*/*v*) by vortexing for 1 min followed by ultrasonic extraction for 60 min at room temperature. The extract was then centrifuged at 15,000× *g* for 5 min at 4 °C. For purification, 1 mL of the supernatant was processed using a QuEChERS-PP purification column containing multi-walled carbon nanotubes during the stationary phase [25]. Finally, 10 μL of the purified extract was diluted with methanol–water (5:95, *v*/*v*) to a final volume of 1 mL. The solution was centrifuged at 21,000× *g* for 2 min prior to UPLC-MS/MS analysis.

#### 5.3.2. Instrumental Analysis

Ultra-performance liquid chromatography–tandem mass spectrometry (UPLC-MS/MS) was used to detect toxins in dried specimens of *O. yunnanensis*. Target toxins included: (1) *Omphalotus*-specific toxins (illudin S and M); and (2) common mushroom toxins with gastroenteritis-causing potential (amatoxins: α-amanitin, β-amanitin; muscimol; ibotenic acid; orellanine), based on the clinical symptoms of poisoning incidents. Non-targeted screening was also performed to detect unknown toxins using full-scan MS mode (*m*/*z* 50–500).

The analysis was performed using a Waters ACQUITY I-Class UPLC system (Waters Corporation, Milford, MA, USA) coupled with a Waters Xevo TQ-S triple quadrupole mass spectrometer (Waters, Milford, MA, USA). Chromatographic separation was achieved on an ACQUITY UPLC HSS T3 column (100 mm × 2.1 mm, 1.8 μm; Waters) maintained at 40 °C. The mobile phase consisted of (A) 10 mmol/L ammonium acetate in water and (B) acetonitrile, delivered at a flow rate of 0.3 mL/min using the following gradient program: 0–1 min, 10% B; 1–8 min, 10–95% B; 8–9 min, 95% B; 9–9.1 min, 95–10% B; 9.1–12 min, 10% B for column re-equilibration. The injection volume was 2 μL.

Mass spectrometric detection was performed in positive electrospray ionization (ESI+) mode with multiple reaction monitoring (MRM). The optimized MS parameters were as follows: capillary voltage, 2.0 kV; source temperature, 150 °C; desolvation temperature, 500 °C; desolvation gas flow, 900 L/h; and cone gas flow, 150 L/h. The MRM parameters for the target toxins, including illudins S and M, are summarized in Table 2 [30].

#### 5.3.3. Method Validation and Quantification

The method was rigorously validated according to recognized guidelines for analytical method validation [11,25]. The analytical standards of illudin S (purity ≥ 98%, CAS: 28276-28-0) and illudin M (purity ≥ 97%, CAS: 28276-27-9) used for calibration and validation were purchased from Sigma-Aldrich (Sigma-Aldrich Corporation, St. Louis, MO, USA) with product numbers 16458 and 16459, respectively. Matrix-matched calibration curves were established using blank *Lentinula edodes* samples spiked with standard solutions of the two toxins at seven concentration levels (0.5–200 μg/L for most analytes). The authenticity and purity of the standards were verified by comparing their retention times, mass spectra, and fragmentation patterns with those reported in previous studies [9,11,14] and the manufacturer’s certification. Linear regression analysis demonstrated excellent linearity with correlation coefficients (R^2^) greater than 0.995 for all target toxins. The limits of detection (LOD) and quantification (LOQ) were determined as signal-to-noise ratios of 3 and 10, respectively. Recovery experiments were performed by spiking blank samples at three concentration levels (10, 20, and 100 mg/kg) with six replicates at each level. The mean recoveries ranged from 85.2% to 106.3%, with relative standard deviations (RSDs) less than 12.5%. Quantification was performed using the external standard method with matrix-matched calibration curves to compensate for matrix effects.

## Figures and Tables

**Table 1:** *Omphalotus yunnanensis* illudin S content and collection information.

Specimen Voucher	Collection Date	Location	Illudin S Content (mg/kg, Dry Weight)
YNHK20230717-01	17 July 2023	China: Yunnan	6.98 mg/kg
YNHHXJCX20231015-01	15 October 2023	China: Yunnan	28.9 mg/kg
HN4600420250118-01	18 January 2025	China: Hainan	86.1 mg/kg

**Table 2:** Optimized MRM parameters for mushroom toxin detection.

Analyte	Precursor Ion (*m*/*z*)	Product Ion (*m*/*z*)
Illudin S	247.1	201.1/229.1
Illudin M	231.1	213.1/203.1

## Data Availability

The original contributions presented in this study are included in the article. Further inquiries can be directed to the corresponding author.
